# *CHI3L1* polymorphisms, cord blood YKL-40 levels and later asthma development

**DOI:** 10.1186/s12890-016-0239-8

**Published:** 2016-05-18

**Authors:** Jakob Usemann, Urs Frey, Ines Mack, Anne Schmidt, Olga Gorlanova, Martin Röösli, Dominik Hartl, Philipp Latzin

**Affiliations:** University of Basel Children’s Hospital, University of Basel, Basel, 4056 Switzerland; Division of Respiratory Medicine, Department of Paediatrics, Inselspital, Bern University Hospital, University of Bern, Bern, 3010 Switzerland; Swiss Tropical and Public Health Institute Basel, Basel, 4051 Switzerland; University of Basel, Basel, 4003 Switzerland; Children’s Hospital, University of Tuebingen, Tuebingen, 72076 Germany

**Keywords:** Asthma, *CHI3L1* protein, Children, Cohort study, Cord blood, Genetic association study, Genetic variation, Infants, YKL-40 protein

## Abstract

**Background:**

Single nucleotide polymorphisms (SNPs) in chitinase 3-like 1 (*CHI3L1*), the gene encoding YKL-40, and increased serum YKL-40 levels are associated with severe forms of asthma. It has never been addressed whether SNPs in *CHI3L1* and cord blood YKL-40 levels could already serve as potential biomarkers for milder forms of asthma. We assessed in an unselected population whether SNPs in *CHI3L1* and cord blood YKL-40 levels at birth are associated with respiratory symptoms, lung function changes, asthma, and atopy.

**Methods:**

In a prospective birth cohort of healthy term-born neonates (*n* = 260), we studied *CHI3L1* polymorphisms, and measured cord blood YKL-40 levels by ELISA in (*n* = 170) infants. Lung function was performed at 5 weeks and 6 years. Respiratory health during the first year of life was assessed weekly by telephone interviews. Diagnosis of asthma and allergic sensitisation was assessed at 6 years (*n* = 142).

**Results:**

The SNP rs10399805 was significantly associated with asthma at 6 years. The odds ratio for asthma was 4.5 (95 % CI 1.59–12.94) per T-allele. This finding was unchanged when adjusting for cord blood YKL-40 levels. There was no significant association for cord blood YKL-40 levels and asthma. SNPs in *CHI3L1* and cord blood YKL-40 were not associated with lung function measurements at 5 weeks and 6 years, respiratory symptoms in the first year, and allergic sensitisation at 6 years.

**Conclusion:**

Genetic variation in *CHI3L1* might be related to the development of milder forms of asthma. Larger studies are warranted to establish the role of YKL-40 in that pathway.

**Electronic supplementary material:**

The online version of this article (doi:10.1186/s12890-016-0239-8) contains supplementary material, which is available to authorized users.

## Background

The incidence of childhood asthma is increasing [1], and early identification of infants at risk could help in the prevention and treatment of this disease. Several studies have proposed that the recently discovered biomarker YKL-40 could be useful in the diagnosis of asthma [2]. The chitinase-like protein, YKL-40, is secreted by macrophages, neutrophils and epithelial cells: particularly in people with severe asthma [3, 4]. In serum, YKL-40 was elevated in children and adults with severe asthma [5–7], and inversely correlated with lung function measures [6, 7]. It was recently suggested that YKL-40 could be involved directly in airway remodeling [4, 5, 8]. Genetic studies revealed that variation in the gene encoding YKL-40, chitinase 3-like 1 (*CHI3L1*), contributes to the pathogenesis of asthma [6]. Genetic variation in *CHI3L1* was associated with pathological lung function values in adults [6], and correlated with poor asthma control and inflammatory markers in severe asthmatic children [5].

Direct involvement of YKL-40 in airway remodeling [4, 5, 8] lead us to hypothesise that early measurement of YKL-40 levels might help identify infants at risk for asthma. Until now, only one longitudinal study in high-risk asthmatic children assessed single nucleotide polymorphisms (SNPs) in *CHI3L1* and cord blood YKL-40 levels at birth. The authors identified genotype-specific effects on circulating YKL-40 levels, but no association of SNPs in *CHI3L1* and cord blood YKL-40 with asthma at school age was found [6]. It remains unknown if genetic variations in *CHI3L1* or cord blood YKL-40 levels assessed at birth in unselected infants are associated with milder forms of childhood asthma.

We studied in a birth cohort of unselected infants the association of SNPs in *CHI3L1* and cord blood YKL-40 levels with asthma development. We further assessed the correlation of SNPs in *CHI3L1* and cord blood YKL-40 levels with respiratory symptoms in the first year of life, lung function measures, and allergic sensitisation.

## Methods

Methods are detailed in Additional file 1.

### Study design and subjects

This prospective birth cohort study comprised a group of unselected, healthy neonates recruited antenatally in the region of Bern, Switzerland. The Ethics Committee of the region of Bern approved the study, and written consent from all parents was acquired at enrolment.

### Genotyping and marker selection

**Fig. 1:**
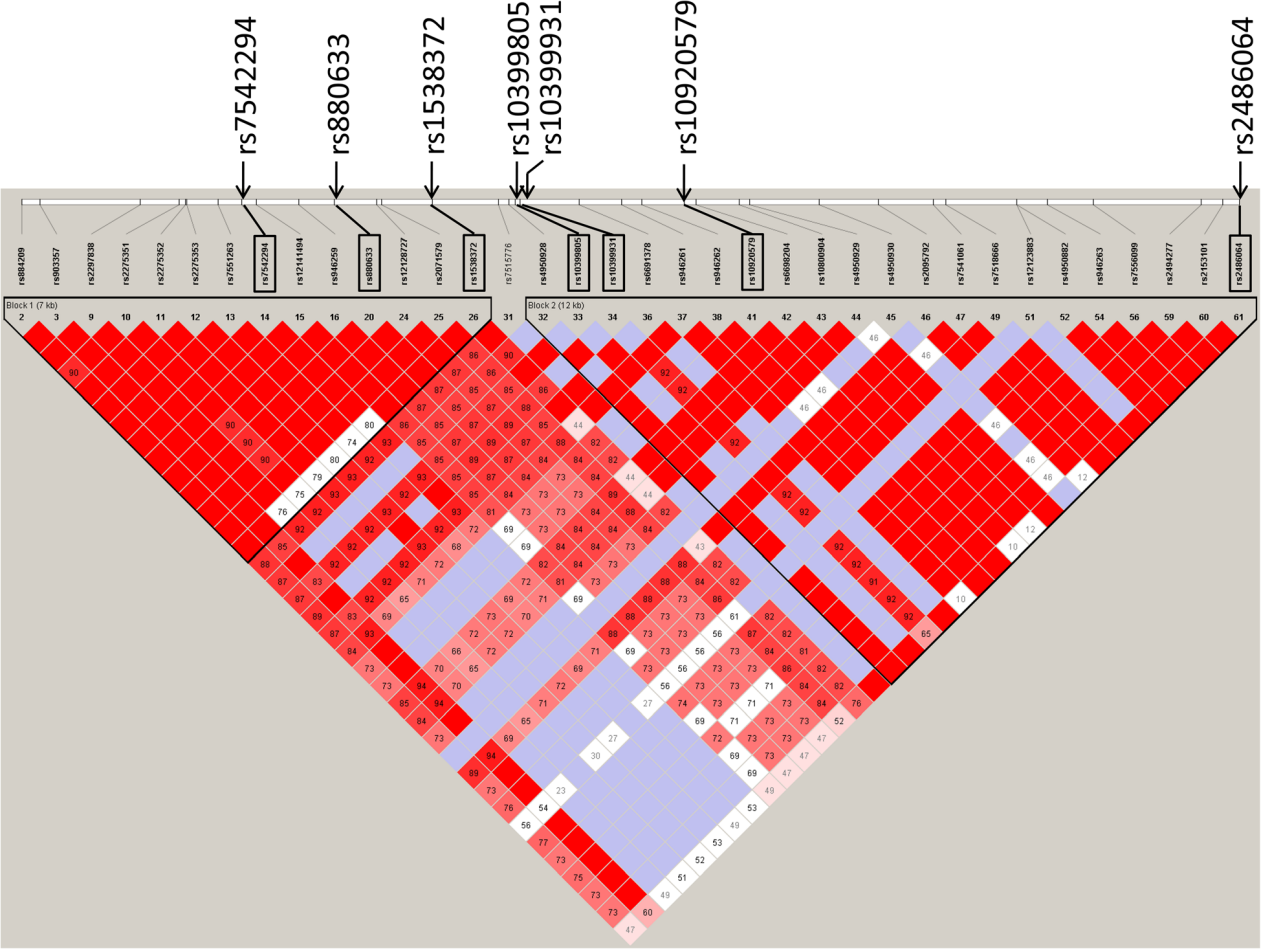
Linkage disequilibrium (LD) map of the *CHI3L1* gene region based on D’ and logarithm of odds [5] score values. D’ values are shown inside each diamond. *Red diamonds* indicate high LD, *pink* (D’ <1 and LOD ≥2), *white* (D’ <1 and LOD <2) and *blue* (D’ =1 and LOD <2). The *black triangles* show the LD-blocks calculated by Haploview. The plot was created using HapMap data (CEU)

Genome-wide SNP genotyping was conducted in collaboration with asthmagene.de (University of Regensburg, Germany) using Illumina HumanOmniExpress Bead Chips (Illumina Inc., San Diego, USA) according to the manufacturer’s instructions. Details on genotyping and quality control methods are given in the online supplement. SNPs from a region 13 kb upstream and 1.2 kb downstream of CHI3L1 were chosen, as previously described [9]. The SNPs selection was done on HapMap CEU data (www.hapmap.org) PhaseII + III Rel28 10^th^ of August 2015, on NCBI B36 assembly, dbSNP b126. Haploview [10] was used to calculate linkage disequilibrium (LD) and to select tagging SNPs with a minor allele frequency (MAF) >5 % and r^2^ > 0.8. In total 7 SNPs were represented on the Chip and included in the analysis (Fig. 1).

### YKL-40 measurement

Cord blood YKL-40 was measured in duplicates by an enzyme-linked immunosorbent assay (ELISA) (R&D Systems, USA). In order to capture the elevated cord blood YKL-40 levels with the limited range of the ELISA, samples had to be pre-diluted 1:20 before measurement. For consistency, all samples were pre-diluted at the same ratios. Data is presented as ng/ml; minimum detection limit of the assay is 3.55 pg/ml.

### Outcomes during the first year

Respiratory symptoms were assessed weekly by telephone interviews [11, 12]. Lung function was performed according to ERS/ATS standards [13]. Tidal volume (V_T_), mean tidal expiratory flow, time to peak tidal expiratory flow (T_PTEF_)/expiratory time (T_E_) ratio and minute ventilation (V’_E_) were measured (Exhalyzer D; Eco Medics AG, Duernten, Switzerland).

### Outcomes at 6 years

Respiratory health was assessed using questions from the International Study of Asthma and Allergies in Childhood [14]. Asthma was diagnosed if one on the following was present in the previous year: (1) physician diagnosis of asthma or (2) episodic wheeze. Atopy was defined by allergic rhinitis, allergic asthma, or atopic dermatitis. A skin-prick test was done for the following allergens: (Dog dander, cat dander, *Dermatophagoides pteronyssinus*, mixed tree pollens, mixed grass pollens, *Alternaria tenuis*, positive control (histamine), negative control (NaCl), Allergomed, Switzerland) positive in case of hives bigger than histamine in any of the tested allergens. Forced expiratory volume in 1 s (FEV_1_), forced vital capacity (FVC) and forced expiratory flow at 25–75 % of FVC (FEF_25–75%_) was measured according to ATS standards [15]. Data are expressed as z-scores using normative data from the Global Lung Function Initiative [16].

### Risk factors

Exposure to pre- and postnatal risk factors [11, 14, 17] on outcomes are given in Additional file 1: Table S1. We validated maternal smoking by cotinine levels in the first urine of the newborn (gas–liquid chromatography, IST, Lausanne, Switzerland). Maternal asthma (self-reported or doctor-diagnosed), maternal atopic disease (history of allergic rhinitis, allergic asthma or atopic dermatitis), and parental education were assessed.

### Statistical analysis

Anthropometric and clinical outcomes were compared with *t*-test and Mann–Whitney *U*-test. We conducted Poisson, logistic and linear regression analysis. For regression models, associations were calculated: (a) unadjusted and, (b) adjusted for known and potential confounders. YKL-40 was categorised in quintiles and associations of YKL-40 levels were calculated using the Cochran-Armitage trend test, shown as *P*_trend_. A trend of association was defined for *P*_trend_ <0.2. We used an additive genetic model and corrected for multiple comparisons according to Benjamini-Hochberg [18]. A *P*-value <0.05 was considered significant. Data was analysed with STATA®, R [19], PLINK [20]. Power was calculated with Quanto [21].

## Results

From 1999 to 2007 the study enrolled *n* = 260 infants with genotyping performed in *n* = 225. After genetic quality control and exclusion of individuals without YKL-40 measurements, *n* = 170 remained for analysis. Of these, *n* = 28 children were lost to follow-up resulting in *n* = 142 school-aged children (Additional file 1: Figure S1). Population characteristics and clinical outcomes are given in Table 1 (equally distributed by sexes), and possible risk factors in Additional file 1: Table S1. Characteristics of the SNPs are shown in Additional file 1: Table S2. None of the SNPs were associated with cord blood YKL-40 levels (Additional file 1: Table S3).

**Table 1:** Population characteristics and distribution of clinical outcomes

	Mean ± S.D.	Median (IQR)	Range	N (%)
Anthropometrics at birth^a^				
Gestational age weeks	39.7 ± 1.1	40 (39.1–40.7)	37.0–42.0	
Weight kg	3.4 ± 0.4	3.4 (3.1–3.7)	2.2–4.9	
Length cm	49.5 ± 1.9	50.0 (48–51)	45–55	
Anthropometrics at follow-up^a^				
Age years	6.0 ± 0.3	6 (5.9–6.2)	5.1– 6.9	
Weight kg	22.7 ± 3.8	22.2 (20.0–24.5)	16–35.8	
Length cm	117.5 ± 5.6	117.5 (114–121)	(104–107)	
YKL-40 in cord blood^a^				
YKL-40 ng/ml	42.4 ± 28.0	43.5 (23.3–63.3)	0–97.2	
Clinical outcomes during the first year^a^				
Wks with daytime resp. sympt.	4.6 ± 4.6	3 (1–7)	0–24	
Wks with nighttime resp. sympt.	3.8 ± 3.8	3 (1–6)	0–21	
Wks with severe daytime resp. sympt.	0.5 ± 0.9	0 (0–1)	0–5	
Wks with severe nighttime resp. sympt.	0.6 ± 1.1	0 (0–1)	0–8	
Lung function at 5 weeks^a^				
Tidal volume ml	32.5 ± 5.5	32.5 (28.1–36.4)	21–51	
Mean tidal expiratory flow	43.3 ± 10.4	41.6 (35.7–49.3)	21–79	
T_PTEF_/T_E_	36.2 ± 10.8	34.7 (28.7–41.8)	16–73	
Minute Ventilation ml · min^−1^	1427 ± 270	1405 (1239–1586)	870–2333	
Clinical data at 6 years				
Asthma^b^				15 (12 %)
Atopy^c^				53 (39 %)
Positive prick test^d^				18 (17 %)
Lung function at 6 years				
FVC z-score^e^	−0.41 ± 0.98	−0.04 (−1.18–0.32)	−2.25–2.02	
FEV_1_ z-score^f^	−0.09 ± 0.96	−0.21 (−0.83–0.73)	−0.25–2.02	
FEF_25–75%_ z-score^g^	−0.04 ± 0.94	−0.04 (−0.68–0.55)	−2.95–2.27	

*FEV*_*1*_ Forced expiratory volume in 1 s, *FVC* Forced vital capacity, *FEF*_*25–75%*_ Forced expiratory flow at 25–75 % of FVC, *IQR* Interquartile range, *N* Number, *S.D.* Standard deviation, *Wks* Weeks, *resp. sympt* Respiratory symptoms. Data are given as mean (S.D.) median (IQR) or number (percentage) of infants. ^a^Data on = 142 infants. ^b^Missing data on *n* = 7; data available for *n* = 135. ^c^Missing data on *n* = 4; data available for *n* = 138. ^d^Missing data on *n* = 38; data available for *n* = 104. ^e^Missing data on *n* = 51; data available for *n* = 91. ^f^Missing data on *n* = 66; data available for *n* = 76. ^g^Missing data on *n* = 62; data available for *n* = 80

### Outcomes during the first year

SNPs and cord blood YKL-40 levels were neither associated with ‘any respiratory symptoms’ nor with ‘severe respiratory symptoms’ during the first year of life in the univariable analysis. When adjusting for potential confounders on respiratory symptoms [12] this association remained non-significant (Additional file 1: Table S4). Associations between SNPs and cord blood YKL-40 with lung function at 5 weeks are given in Additional file 1: Table S5. There was no association in either the univariable or adjusted models for any of the examined parameters. Sensitivity analysis with adjustment for e.g. maternal asthma and delivery type revealed similar results (data not shown).

### Outcomes at 6 years

**Fig. 2:**
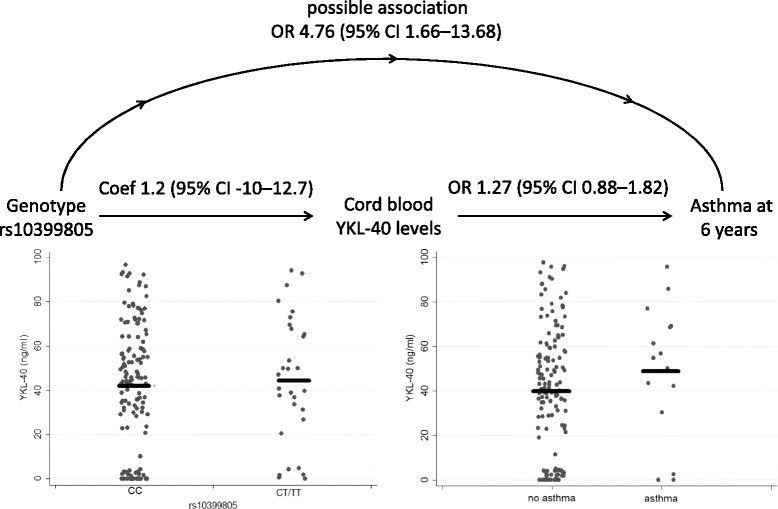
Schematic presentation of associations of genetic polymorphisms in the *CHI3L1* gene (rs10399805) and cord blood YKL-40 levels with asthma at 6 years. Coef: coefficient; CI: confidence interval; OR: odds ratio. There was no association of rs10399805 with cord blood YKL-40 levels, nor an association between YKL-40 and asthma at 6 years. In the unadjusted analysis, the SNP rs10399805 was associated with asthma at 6 years, even after adjustment for YKL-40 levels (Table 3). The continuous line represents the mean. Data are derived from *n* = 135 children with *n* = 15 with asthma diagnosis

At follow-up, there were 76 (53 %) males, 15 (11 %) asthmatics, 18 (17 %) had a positive prick test and 53 (39 %) were atopic. The SNP rs10399805 [T] was significantly associated with asthma at 6 years (Benjamini-Hochberg adjusted *P* = 0.031). The odds ratio (OR) for asthma was 4.5 (95 % CI 1.59–12.94) per T-allele in the univariable assocation. This study was sufficiently powered (80 %) at the 5 % level of significance for an OR >4. When adjusting for maternal atopy, parental smoking and parental education, this association was non-significant (Table 2). A protective effect of the A-allele of rs10399931 for asthma and atopy in the adjusted model [OR asthma 0.21 (95 % CI 0.04–0.98), OR atopy 0.43 (95 % CI 0.21–0.86)] was observed. These results were non-significant when adjusting for multiple comparisons (Table 2). Since asthma is known to be associated with YKL-40 levels (5, 6), we adjusted the associations of SNPs with asthma for cord blood YKL-40 levels, which revealed similar results (Table 3). This indicates that YKL-40 does not seem to be involved in the pathway responsible for the observed association between SNPs and asthma (Fig. 2). Positive prick test results were not associated with any of the SNPs in the uni- and multivariable analysis (Additional file 1: Table S6).

**Table 2:** Associations between cord blood YKL-40 levels and SNPs with asthma and atopy at school age

	Univariable association			Multivariable^a^ association		
	OR	95 % CI	*P*-value	OR	95 % CI	*P*-value
Exposure	Outcome asthma^b^					
SNP^*^						
rs10920579	0.25	0.06–1.15	0.305	0.23	0.05–1.2	0.365
rs880633	1.33	0.63–2.89	0.777	1.05	0.46–2.38	0.901
rs10399931	0.21	0.05–0.95	0.212	0.21	0.04–0.98	0.287
rs10399805	4.50	1.59–12.94	0.031	3.42	1.12–10.56	0.224
rs1538372	0.74	0.31–1.72	0.777	0.68	0.28–1.63	0.901
rs7542294	2.80	1.29–7.01	0.158	2.37	0.87–6.46	0.365
rs2486064	0.89	0.42–1.91	0.777	0.94	0.42–2.19	0.901
YKL-40 (ng/ml)						
YKL-40 non-detects	1	reference	*P*_trend_	1	reference	*P*_trend_
YKL-40 (7–37.9)	0.86	0.13–5.54		0.82	0.12–5.67	
YKL-40 (38–49.9)	1.21	0.22–6.43		1.45	0.24–8.61	
YKL-40 (50–65.9)	1.72	0.35–8.38		2.27	0.41–12.2	
YKL-40 (66–98)	2.24	0.49–10.24	0.218^**^	2.52	0.49–13.01	0.169^**^
Exposure	Outcome atopy^c^					
SNP^*^						
rs10920579	0.56	0.26–1.14	0.691	0.50	0.24–1.05	0.714
rs880633	0.92	0.55–1.51	0.96	0.94	0.57–1.56	0.913
rs10399931	0.45	0.24–0.91	0.169	0.43	0.21–0.86	0.251
rs10399805	1.43	0.64–3.19	0.96	1.63	0.71–3.75	0.913
rs1538372	0.83	0.48–1.39	0.96	0.81	0.47–1.39	0.913
rs7542294	1.25	0.62–2.55	0.96	1.37	0.66–2.81	0.913
rs2486064	0.98	0.61–1.59	0.96	0.89	0.54–1.47	0.913
YKL-40 (ng/ml)						
YKL-40 non-detects	1	reference	*P*_trend_	1	reference	*P*_trend_
YKL-40 (7–37.9)	1.15	0.42–3.13		1.09	0.43–3.21	
YKL-40 (38–49.9)	1	0.37–2.67		0.98	0.37–2.71	
YKL-40 (50–65.9)	1.44	0.54–3.83		1.35	0.55–3.91	
YKL-40 (66–98)	1.76	0.67–4.61	0.188^**^	1.69	0.66–4.58	0.232^**^

*CI* Confidence interval, *OR* Odds ratio. *SNP* Single nucleotide polymorphism. Cord blood YKL-40 levels are compared with YKL-40 non-detects. ^a^Adjusted for the following additional risk factors: sex, parental smoking during childhood, maternal atopy, parental education. ^b^Asthma was diagnosed if one on the following was present in the previous year: (1) physician diagnosis of asthma or (2) episodic wheeze. Missing data on *n* = 7; data available for *n* = 135. ^c^Defined if one of the following was present: asthma, allergic rhinitis, atopic eczema or positive prick test. Missing data on *n* = 4; data available for *n* = 138. ^*^ *P-*values for SNPs are shown after correction for multiple testing according to Benjamini-Hochberg. ^**^ *P*_trend_-values were calculated with the Cochran-Armitage trend test

**Table 3:** Associations between SNPs and asthma at school age adjusted for cord blood YKL-40 levels

	Univariable association			Univariable association adjusted for YKL-40 levels		
	OR	95 % CI	*P*-value	OR	95 % CI	*P*-value
Exposure	Outcome asthma^a^					
SNP^*^						
rs10920579	0.25	0.06–1.15	0.305	0.23	0.05–1.08	0.254
rs880633	1.33	0.63–2.89	0.777	1.27	0.59–2.75	0.654
rs10399931	0.21	0.05–0.95	0.212	0.2	0.04–0.89	0.189
rs10399805	4.50	1.59–12.94	0.031	4.76	1.66–13.62	0.026
rs1538372	0.74	0.31–1.72	0.777	0.71	0.31–1.68	0.654
rs7542294	2.80	1.29–7.01	0.158	2.66	1.06–6.72	0.189
rs2486064	0.89	0.42–1.91	0.777	0.84	0.39–1.81	0.654

*CI* Confidence interval, *OR* Odds ratio. *SNP* Single nucleotide polymorphism. ^a^Asthma was diagnosed if one on the following was present in the previous year: (1) physician diagnosis of asthma or (2) episodic wheeze. Missing data on *n* = 7; data available for *n* = 135. ^*^ *P-*values for SNPs are shown after correction for multiple testing according to Benjamini-Hochberg

**Fig. 3:**
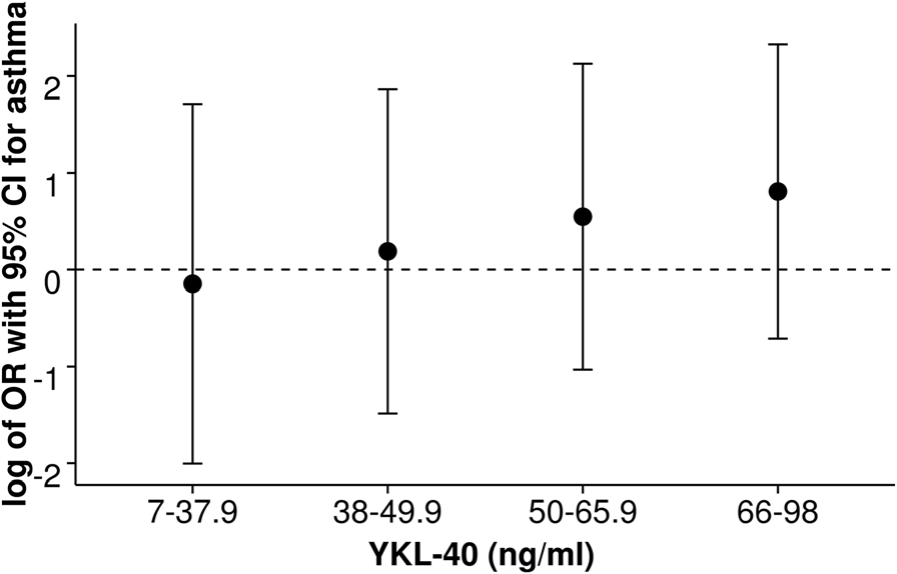
Associations between cord blood YKL-40 levels and asthma at 6 years. CI: confidence interval; log: logarithmic; OR: odds ratio. Data is given as OR (with 95 % CI) for asthma at 6 years for 4 different categories of cord blood YKL-40 levels above detection limit. YKL-40 non-detects (not shown) served as reference. Data are derived from *n* = 135 children with *n* = 15 with asthma diagnosis. The combined *P*–value for trend for the adjusted OR was (P_trend_ = 0.169)

We observed a trend of association of YKL-40 levels with the OR for asthma and positive prick test results (*P*_trend_ = 0.169) (Table 2, Fig. 3 and Additional file 1: Table S6). Associations of SNPs and cord blood YKL-40 levels with lung function measures are given in Additional file 1: Table S7. There was no association in the univariable or adjusted models for any of the examined parameters.

## Discussion

### Summary

In this cohort study, we examined the effect of genetic variation of *CHI3L1* and cord blood YKL-40 levels of unselected infants. We demonstrate that genetic variation of *CHI3L1* is associated with asthma in early childhood. In particular, the T-allele of rs10399805 significantly increased the risk for asthma, even after correction for multiple testing. Cord blood YKL-40 levels were not associated with any of the investigated outcomes.

### Limitations and strenghts

The main limitation is the small sample size, which restricts power to identify weak associations. However, in contrast to previous cross-sectional studies [5, 9, 22, 23], this study was conducted in a cohort with a highly elaborate design, which naturally limits sample size. Unlike a previous prospective study [6], we diagnosed asthma based on one definition and assessed further outcomes on respiratory health (lung function at 5 weeks and 6 years, respiratory symptoms during the first year). Nevertheless, most of the correlations in this study were negative and only some findings indicated a trend of association. Trends of associations might be rendered to significant findings with more asthma cases. In an unselected population, this could be achieved by increasing the sample size or by studying a high-risk population. Both options have their drawbacks since larger birth cohort studies with a similar elaborate design are lacking, while studies in high-risk populations are predominantly multi-center studies, introducing a bias on asthma diagnosis due to different study sites.

Associations between genetic variation in *CHI3L1* and asthma are controversial in the discussion, and the SNP rs4950928 has been extensively studied in this context. While a study in Korean children found no association of rs4950928 with asthma [23], other studies reported conflicting findings on the suspected risk allele of rs4950928 and asthma [6, 9, 22]. In a Danish study of adults, Rathcke et al. [22] found that homozygosity of the G allele was associated with asthma, while Ober et al. [6] found a higher prevalence of the C allele to be associated with asthma in three different populations (genetically related subjects, children recruited in Germany and a mixed population of children and adults). Interestingly, in a study performed in adults at increased risk for asthma [9], the rs4950928 polymorphism was not associated with airflow obstruction, indicating that severity of asthma is not modified by this polymorphism. It is a weakness that rs4950928 was not genotyped in our study and that we cannot conclude on associations between this extensively studied tag SNP in *CHI3L1* and asthma development. However, we investigated rs10920579, which was in high linkage disequilibrium with the SNP rs4950928 (*r*^2^ = 1), and therefore serves as a good tagging SNP for the genetic variation associated with asthma at this locus. Moreover, we performed analysis of associations with 6 other SNPs in the *CHI3L1* gene region, and these results support the overall conclusions of our study. Nevertheless, we are unable to determine if the effect of genetic variation of *CHI3L1* on current asthma is reflected by current YKL-40 levels, as YKL-40 levels were not measured at 6 years.

A major strength is our use of a rigorous methodology in the assessment of respiratory symptoms and lung function measurements. We studied the association of genetic variation of *CHI3L1* and cord blood YKL-40 levels with respiratory morbidity at various time points in unselected infants. Applicability of these results is not limited to high-risk subjects, as investigated previously [6], but extend as well to the general population.

The observed asthma prevalence of 11 % in this study represents well the general population prevalence in Switzerland, as reported from larger cross-sectional studies [24]. Most of the asthmatics in this study belong to the episodic wheezing phenotype, and multiple trigger wheezing phenotypes are rarely found in this cohort of unselected infants. Diagnosing asthma in paediatric subjects is challenging, resulting in underdiagnosed cases [24]. Even though rigorous assessment of asthma diagnoses by study physicians was performed in this cohort with validated questionnaires [25], there remains a risk that some asthmatics were not correctly identified. Since the observed prevalence of asthmatic subjects is in concordance with the published prevalence in this region [24], we however consider the number of false negative diagnoses to be low.

### Comparison with literature

#### SNPs in *CHI3L1* and cord blood YKL-40 levels

We did not find any associations of the investigated SNPs with cord blood YKL-40 levels. This contradicts previous publications, describing an association of rs4950928 with cord blood YKL-40 levels (6). Interestingly, in contrast to previous publications (6), we had 21 % measurements below detection limit. The different distribution of YKL-40 levels in our cohort of unselected infants compared to YKL-40 levels in high-risk asthmatic subjects (6) might account for the lack of association of genetic variation of *CHI3L1* and cord blood YKL-40 levels. This hypothesis, however, requires validation in a larger sample.

### Respiratory symptoms and asthma

There was no association of any of the investigated SNPs and cord blood YKL-40 levels with respiratory symptoms during the first year, a subject that has not been addressed in previous studies. The association of genetic variation of *CHI3L1* and asthma, however, has been well documented in large European populations studies [4, 9], while other authors suggested no association of polymorphisms in *CHI3L1* with asthma [26].

We identified a thus far unreported association between rs10399805 and asthma at 6 years. Moreover, rs10399805 was not associated with cord blood YKL-40 levels (Additional file 1: Table S3) and cord blood YKL-40 levels did not modify the association of rs10399805 with asthma (Table 3). Thus, from our data we speculate that rs10399805 is more relevant for asthma development than cord blood YKL-40 levels in unselected infants (Fig. 2). When adjusting for potential risk factors, this association did not reach statistical significance anymore, indicating other risk factors besides rs10399805 to also be relevant for disease development. The protective effect of rs10399931[A] for asthma at 6 years, although non-significant after adjusting for multiple testing (Table 2), provided further evidence for the relevance of genetic variation in *CHI3L1* for asthma development. This SNP was reportedly associated with asthma in Taiwanese adults [27].

We observed a trend of association of increased YKL-40 levels with the OR for asthma at 6 years (Fig. 3). We speculate that the low number of asthmatic subjects in this study of unselected infants resulted in increasingly large confidence intervals and, hence, our findings did not reach formal significance level (*P*_trend_ = 0.169). An association of increased serum YKL-40 levels with severe asthma in adult [6, 7] and paediatric subjects [5] has been reported. Recent studies regarded YKL-40 as not only a simple biomarker for asthma, but as directly involved in airway remodeling. Increased YKL-40 levels were associated with bronchial wall thickening on computerised tomography in children with severe asthma [5] and subepitehlial basement membrane thickness in adults [4]. Mechanistically, Bara et al. showed that YKL-40 increased bronchial smooth muscle cell proliferation [8]. Despite emerging evidence for a direct involvement of YKL-40 in asthma development, in this study of unselected infants, YKL-40 in cord blood did not serve as a predictor for asthma in early childhood. Future studies will need to investigate if this was due to the low level of asthmatics in the general population and in our cohort.

### Atopy

We identified a protective effect of the SNP rs10399931[A] with atopy and positive prick test results, although non-significant after correction for multiple testing. Several studies investigated the association of genetic variation in *CHI3L1* with allergic diseases, but the SNP rs10399931 has not been reported in that context. An association of rs10399805 with atopy, recently reported in Korean children (22), could not be confirmed in our study, possibly due to differing study populations.

While there was no association of YKL-40 with atopy and in two large population studies [4, 6], there was an association of higher serum YKL-40 with allergic rhinitis [28]. In our study, we observed a trend of association of increased YKL-40 levels with the OR for atopy and positive prick (*P*_trend_ = 0.188, *P*_trend_ = 0.128). In conclusion, we observed an association of genetic variation in *CHI3L1* and cord blood levels YKL-40 with allergic diseases, but due to low sample size, this association did not reach statistical significance.

### Lung function

We did not find an association of genetic variation in *CHI3L1* and YKL-40 levels in cord blood with lung function measures at 5 weeks and 6 years. An association of lung function measures with genetic variation in *CHI3L1* and YKL-40 levels has been well described. Genetic variation in *CHI3L1* was associated with FEV_1_, FVC and FEV_1_/FVC–ratios in adult European and Taiwanese populations [4, 9, 27] and serum YKL-40 levels were inversely associated with FEV_1_ in adult [4, 6, 7], but not paediatric asthmatics [5]. We conclude from these findings that in contrast to previous cross-sectional studies, genetic variation in *CHI3L1* and cord blood YKL-40 levels at birth are not associated with lung function measures in unselected infants.

### Relevance

From cross-sectional studies it is well known that both, genetic variation in *CHI3L1* and YKL-40 levels are associated with asthma, atopy, and lung function measures. Our study further explores this association since we assessed genetic variation in *CHI3L1* and YKL-40 in cord blood within the setting of a cohort study before the onset of these diseases. Moreover, our study is the first to study this association in unselected, healthy infants. The limited number of diseased subjects in the general population and in this cohort study might be the cause of the non-significant associations of YKL-40 with asthma, atopy, and prick test results. Studying this association in a much larger cohort might result in significant findings and reveal further insights for the relevance of YKL-40 for these diseases.

## Conclusion

In this prospective cohort study we found no robust association between genetic variation in *CHI3L1* and asthma development, but found some indication that rs10399805 might be related to asthma diagnosis at 6 years. In order to replicate findings of this study, and to investigate its robustness, larger studies with a prospective design in an unselected population are warranted. The trend of association between elevated cord blood YKL-40 levels and asthma requires further validation before YKL-40 may be considered an early biomarker for asthma development in unselected infants.

### Availability of data

Authors would be pleased to consider requests to share original study data.

## Additional file

Additional file 1: Data S1. Methods. **Figure S1.** Flow chart of the study population. **Table S1.** Potential risk factors of the study subjects. **Table S2.** Characteristics and prevalence of the SNPs employed in this study. **Table S3.** Associations between SNPs and cord blood YKL-40 levels. **Table S4.** Associations between SNPs and cord blood YKL-40 levels with weeks with any respiratory symptoms and weeks with severe respiratory symptoms during the first year of life. **Table S5.** Associations between SNPs and cord blood YKL-40 levels with lung function at 5 weeks. **Table S6.** Associations between SNPs and cord blood YKL-40 levels with positive prick test at school age. **Table S7.** Associations between SNPs and cord blood YKL-40 levels with lung function at 6 years. (DOCX 197 kb)

## Abbreviations

CHI3L1: chitinase-3-like-1
ELISA: enzyme-linked immunosorbent assay
FEF_25-75%_: forced expiratory flow at 25-75% of FVC
FEV_1_: forced expiratory volume in 1 second
FVC: forced vital capacity
LD: linkage disequilibrium
MAF: minor allele frequency
LOD: logarithm of odds
V’_E_: minute ventilation
SNPs: single nucleotide polymorphisms
T_E_: expiratory time
T_PTEF_: time to peak tidal expiratory flow
V_T_: tidal volume
YKL-40: A 40 kilodalton chitinase-like protein named after the first three N-terminal amino; acids tyrosine (Y), lysine (K) and leucine (L)
