# Supplementary material for: CHI3L1 polymorphisms, cord blood YKL-40 levels and later asthma development
Source: BMC Pulm Med. 2016 May 18;16:81. doi: 10.1186/s12890-016-0239-8 (PMC4870763; doi:10.1186/s12890-016-0239-8)
Supplement: Additional file 1: Data S1. — Methods. Figure S1. Flow chart of the study population. Table S1. Potential risk factors of the study subjects. Table S2. Characteristics and prevalence of the SNPs employed in this study. Table S3. Associations between SNPs and cord blood YKL-40 levels. Table S4. Associations between SNPs and cord blood YKL-40 levels with weeks with any respiratory symptoms and weeks with severe respiratory symptoms during the first year of life. Table S5. Associations between SNPs and cord blood YKL-40 levels with lung function at 5 weeks. Table S6. Associations between SNPs and cord blood YKL-40 levels with positive prick test at school age. Table S7. Associations between SNPs and cord blood YKL-40 levels with lung function at 6 years. (DOCX 197 kb) [file 12890_2016_239_MOESM1_ESM.docx]

**Online supplement**

***CHI3L1* polymorphisms, cord blood YKL-40 levels and later asthma development**

Jakob Usemann^1,2^, Urs Frey^1^, Ines Mack^1^, Anne Schmidt^1,2^, Olga Gorlanova^1^, Martin Röösli^3,4^, Dominik Hartl^5*^ and Philipp Latzin^1,2 *^

^1^ University Children`s Hospital Basel, Basel, Switzerland

^2^ Division of Respiratory Medicine, Department of Paediatrics, Inselspital, Bern University Hospital, University of Bern, Switzerland

^3^ Swiss Tropical and Public Health Institute Basel, Basel, Switzerland

^4^ University of Basel, Basel, Switzerland

^5^ Children’s Hospital, University of Tuebingen, Tuebingen, Germany

**S1 Methods**

**Study design and subjects**

In this prospective cohort study, healthy term neonates were recruited antenatally from April 1999 to January 2007 in the region of Bern, Switzerland, described in detail elsewhere [[1](#_ENREF_1)]. Exclusion criteria for the study were pre-term delivery (<37 weeks), major birth defects, respiratory distress after birth, other significant perinatal disease or later diagnosis of airway malformation or specific chronic respiratory disease [[1-3](#_ENREF_1)]. The Ethics Committee of the region of Bern approved the study and written consent from all parents was acquired at enrolment.

**Genotyping and quality control**

DNA was extracted from umbilical cord blood. Genome-wide SNP genotyping was performed by Illumina HumanOmniExpress Bead Chips (Illumina Inc., San Diego, USA). Individuals with low genotype rate (<97%), unusual high or low heterozygosity (±3 SD beyond the mean) and discordance between reported and observed sex were excluded (see Figure 1). In total, 142 unrelated individuals of European ancestry with measured YKL-40 levels were considered for the analysis. SNPs were excluded if they had a call rate less than 98%, minor allele frequency (MAF) more than >5%, and Hardy-Weinberg equilibrium p-value less than 10^-6^. Quality control statistics was calculated using PLINK [[4](#_ENREF_4)] version 1.07 and R version 3.0.2 [[5](#_ENREF_5)] (www.r-project.org) using the GenABEL package.

**YKL-40 measurement**

Cord blood was acquired from the umbilical cord and stored immediately at –80°C. Serum was diluted 1:20 before YKL-40 was measured in duplicates by a sandwich enzyme-linked immunosorbent assay (ELISA) kit (R&D Systems, USA) according to the manufacturer´s instructions. The mean of the 2 duplicates was used in the statistical analysis. Minimum detection limit of the assay is 3.55 pg/ml, inter-assay variability is 5.3%, and intra-assay variability is 14%. Data are presented as ng/ml.

**Outcomes during the first year**

The outcome respiratory symptoms during the first year were assessed by weekly telephone interviews done by research nurses using a standardised symptom score that groups symptoms into four levels according to severity, with a high sensitivity for lower respiratory tract symptoms [[3](#_ENREF_3), [6](#_ENREF_6)]. The outcomes ‘weeks with respiratory symptoms’ were defined as total number of weeks a child had any respiratory symptom, independent of type or severity; ‘weeks with severe respiratory symptoms’ were defined as a symptom score ≥3 e.g repeated sleep disturbances during the night, or general practitioner consultation, as described previously [[3](#_ENREF_3)]. Lung function was performed according to ERS/ATS standards [[7](#_ENREF_7)] during unsedated sleep in supine position with the head midline, as previously reported [[8](#_ENREF_8)]. For infant lung function at 5 weeks, we used the first 100 regular breaths of tidal breathing during non-rapid eye movement (non-REM) sleep from the total recording over 10 min. We calculated mean tidal breathing parameters of flow, volume, and flow-volume loop. Outcome parameters were tidal volume (V_T_), mean tidal expiratory flow, time to peak tidal expiratory flow (T_PTEF_)/expiratory time (T_E_) ratio and minute ventilation (V'_E_). Tidal breathing measurements were done using an ultrasonic flow meter device (Exhalyzer D; Eco Medics AG, Dürnten, Switzerland).

**Outcomes at 6 years**

At follow-up with 6 years, respiratory health was assessed with questions adapted from the International Study of Asthma and Allergies in Childhood (ISAAC) [[9](#_ENREF_9)]. The questionnaires have been validated and used in our study since 1999 [[1](#_ENREF_1)]. Asthma was diagnosed if one on the following was present in the previous year: (1) physician diagnosis of asthma or (2) episodic wheeze. Atopy was defined by allergic rhinitis, allergic asthma or atopic dermatitis. A skin-prick test was done for eight allergens (Dog dander, cat dander, *Dermatophagoides pteronyssinus*, mixed tree pollens, mixed grass pollens, *Alternaria tenuis*, positive control (histamine), negative control (NaCl), Allergomed, Switzerland) positive in case of hives bigger than histamine in any of the tested allergens. Spirometry was performed to measure forced expiratory volume in 1 second (FEV_1_), forced vital capacity (FVC), and forced expiratory flow at 25-75% of FVC (FEF_25-75%_), according to ATS standards [[10](#_ENREF_10)]. Data are expressed as z-scores using normative data from the Global Lung Function Initiative [[11](#_ENREF_11)]. Spirometry was done using the JAEGER^®^ Master-Screen Body (CareFusion, Würzburg, Germany).

**Risk factors**

A standardised questionnaire was employed to assess pre- and post-natal exposure to putative risk factors [[3](#_ENREF_3), [8](#_ENREF_8), [9](#_ENREF_9)] (e.g. siblings, tobacco smoke exposure, and maternal atopy) on the outcomes respiratory symptoms during the first year, at the 5-week lung function, and at 6 years (asthma, atopy, positive prick test and spirometry) (Table S1). We validated maternal smoking by cotinine levels in the first urine of the newborn (gas-liquid chromatography, IST, Lausanne, Switzerland). Maternal asthma (self-reported or doctor-diagnosed asthma) and maternal atopic disease (history of allergic rhinitis, allergic asthma or atopic dermatitis) was assessed. Parental education was categorised into low (<4 years of apprenticeship) and high (≥4 years of apprenticeship).

**Statistical analysis**

Anthropometric and clinical outcomes were compared by sex using *t*-test and Mann-Whitney *U*-test. We performed regression analysis with cord blood YKL-40 levels as outcome and SNPs as exposures. Because 44 (21%) of cord blood YKL-40 levels were below detection limit, we used a Tobit regression model with a detection limit of 1 (concentration ng/ml). To minimise the effect of outliers, and to differentiate between different exposure levels, we categorised YKL-40 (ng/ml) into quintiles: YKL-40 non-detects, YKL-40 (7–37.9), YKL-40 (38–49.9), YKL-40 (50–65.9) and YKL-40 (66–98). The YKL-40 non-detects served as reference level. The overall association of different YKL-40 levels with the outcomes was calculated with the Cochran-Armitage trend test, shown as *P*_trend_. A trend of association was defined for *P*_trend_ <0.2.

We examined the association of SNPs and cord blood YKL-40 levels with respiratory symptoms during the first year. The outcome ‘weeks with respiratory symptoms’ showed an over-dispersion, and data was analysed using Poisson regression based on robust variance estimates [[12](#_ENREF_12)]. First, univariable regression analysis was performed, and secondly, a multivariable model with adjustment for other exposures associated with respiratory symptoms (2) such as sex, gestational weight, and maternal smoking during pregnancy was used. Then, we analysed with univariable linear regression models the associations of SNPs and YKL-40 levels with natural-log-transformed lung function measures at 5 weeks. In a multivariable model we adjusted for anthropometric data (sex, birth length, age and weight at study) and for sensitivity analysis additionally for potential confounders (e.g. maternal asthma, delivery type).

We assessed the associations of SNPs and cord blood YKL-40 levels with the outcomes (asthma, atopy, prick test results) at 6 years with logistic regression. First, univariable analysis was performed for each outcome variable, and secondly, a multivariable model with adjustment for e.g sex and parental smoking was used. Lung function measures at 6 years were analysed with linear regression analysis. First, univariable analysis was performed and secondly with adjustment for presence or absence of siblings, daycare attendance and asthma of the child. For sensitivity analysis we additionally adjusted for potential confounders (e.g. maternal asthma, delivery type).

The selection of variables for the adjusted models was based on physiological plausibility, potential effects of genetic variation on the outcomes and the best fit based on the Akaike information criterion [[13](#_ENREF_13)]. An additive genetic model was used with *P*-values adjusted for multiple testing for 7 tests (since 7 SNPs were used in this study) with the Benjamini-Hochberg procedure (false discovery rate <0.1) [[14](#_ENREF_14)] to balance power and the potential for false-positive results. A *P*–value < 0.05 was considered to be significant. Power was calculated with Quanto [[15](#_ENREF_15)]. Data was analysed with STATA^®^ (Stata Statistical Software: release 13. STATA^®^ Cooperation, College Station, TX, USA), R version 3.0.2 ([www.r-project.org](http://www.r-project.org)) [[5](#_ENREF_5)] using the GenABEL package and PLINK [[4](#_ENREF_4)] version 1.07 (<http://pngu.mgh.harvard.edu/purcell/plink/>).

**References**

1. Fuchs O, Latzin P, Kuehni CE, Frey U: **Cohort profile: the Bern infant lung development cohort**. *Int J Epidemiol* 2012, **41**(2):366-376.

2. Latzin P, Kuehni CE, Baldwin DN, Roiha HL, Casaulta C, Frey U: **Elevated exhaled nitric oxide in newborns of atopic mothers precedes respiratory symptoms**. *American journal of respiratory and critical care medicine* 2006, **174**(12):1292-1298.

3. Latzin P, Frey U, Roiha HL, Baldwin DN, Regamey N, Strippoli MP, Zwahlen M, Kuehni CE, Swiss Paediatric Respiratory Research G: **Prospectively assessed incidence, severity, and determinants of respiratory symptoms in the first year of life**. *Pediatric pulmonology* 2007, **42**(1):41-50.

4. Purcell S, Neale B, Todd-Brown K, Thomas L, Ferreira MA, Bender D, Maller J, Sklar P, de Bakker PI, Daly MJ *et al*: **PLINK: a tool set for whole-genome association and population-based linkage analyses**. *American journal of human genetics* 2007, **81**(3):559-575.

5. Team RDC: **R: A Language and Environment for Statistical Computing**. 2012.

6. Silverman M, Wang M, Hunter G, Taub N: **Episodic viral wheeze in preschool children: effect of topical nasal corticosteroid prophylaxis**. *Thorax* 2003, **58**(5):431-434.

7. Frey U, Stocks J, Coates A, Sly P, Bates J: **Specifications for equipment used for infant pulmonary function testing. ERS/ATS Task Force on Standards for Infant Respiratory Function Testing. European Respiratory Society/ American Thoracic Society**. *The European respiratory journal* 2000, **16**(4):731-740.

8. Fuchs O, Latzin P, Thamrin C, Stern G, Frischknecht P, Singer F, Kieninger E, Proietti E, Riedel T, Frey U: **Normative data for lung function and exhaled nitric oxide in unsedated healthy infants**. *The European respiratory journal* 2011, **37**(5):1208-1216.

9. Asher MI, Keil U, Anderson HR, Beasley R, Crane J, Martinez F, Mitchell EA, Pearce N, Sibbald B, Stewart AW *et al*: **International Study of Asthma and Allergies in Childhood (ISAAC): rationale and methods**. *The European respiratory journal* 1995, **8**(3):483-491.

10. Miller MR, Hankinson J, Brusasco V, Burgos F, Casaburi R, Coates A, Crapo R, Enright P, van der Grinten CP, Gustafsson P *et al*: **Standardisation of spirometry**. *The European respiratory journal* 2005, **26**(2):319-338.

11. Quanjer PH, Stanojevic S, Cole TJ, Baur X, Hall GL, Culver BH, Enright PL, Hankinson JL, Ip MS, Zheng J *et al*: **Multi-ethnic reference values for spirometry for the 3-95-yr age range: the global lung function 2012 equations**. *The European respiratory journal* 2012, **40**(6):1324-1343.

12. Long JS, Freese J: **Regression models for categorical dependent variables using STATA**. In: *Stata Press Publication.* 2014 edn; 2014: 223-241.

13. Akaike H: **A new look at the statistical model identification**. *IEEE Trans Autmat Contr* 1974(19):716-723.

14. Benjamini Y, Hochberg Y: **Controlling the false discovery rate: a practical and powerful approach to multiple testing**. *J Roy Stat Soc B* 1995, **57**:12.

15. **Quanto 1.1: A computer Programm for power and sample size calculations for genetic-epidemiology studies** [<http://biostats.usc.edu/Quanto.html>]


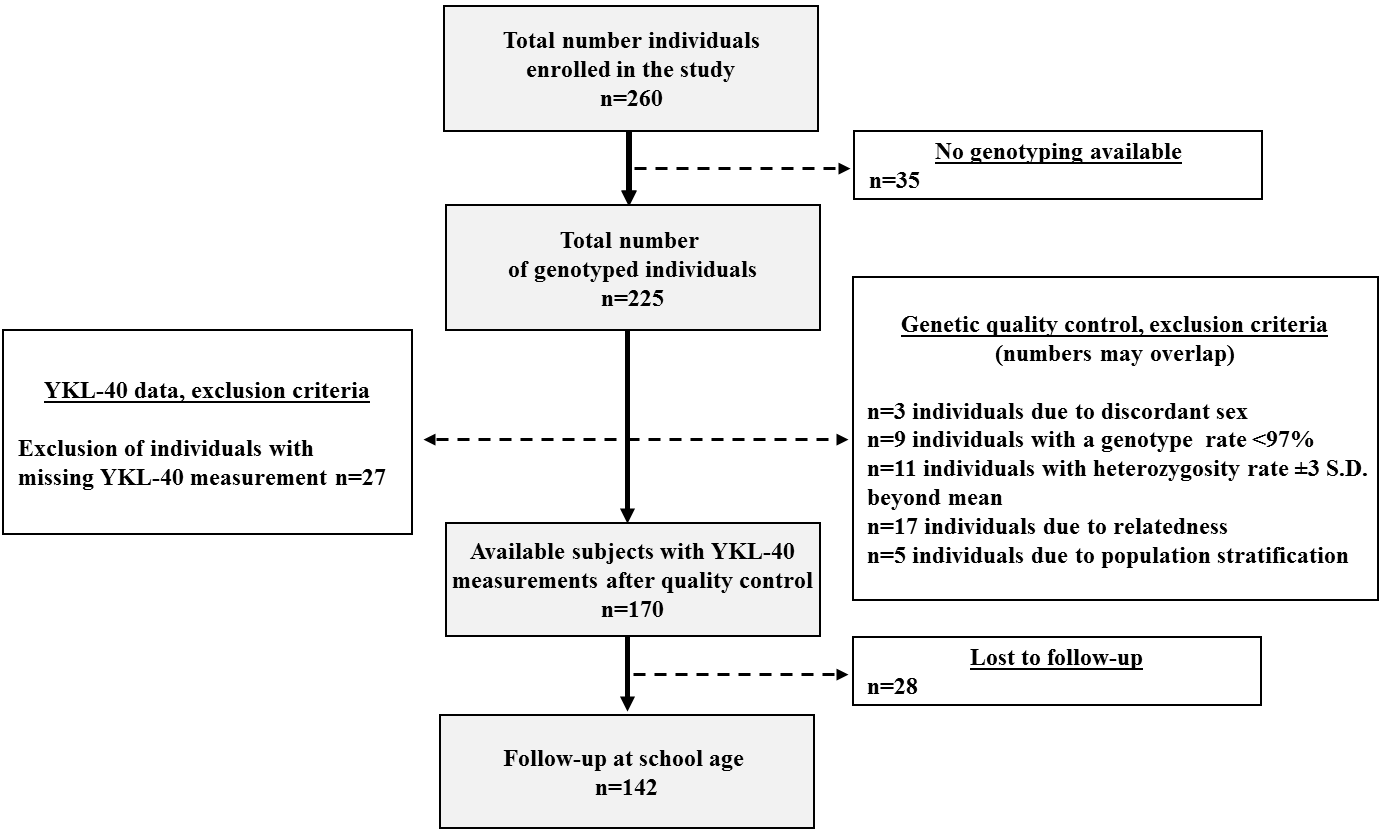


**Figure S1** Flow chart of the study population

| **Table S1 Potential risk factors of the study subjects** | |
| --- | --- |
| Prenatal risk factors | n (%) |
| Male sex | 76 (53.5) |
| Siblings^*^ | 73 (51.5) |
| Caesarean section | 17 (11.9) |
| Maternal asthma^$^ | 17 (11.9) |
| Maternal atopy^#^ | 61 (42.9) |
| Positive maternal skin prick test^+^ | 55 (38.7) |
| Maternal smoking in pregnancy^§^ | 12 (8.5) |
| High maternal education^¶^ | 94 (67.2) |
| Low maternal education^¶^ | 46 (32.9) |
| High paternal education^¶^ | 112 (80) |
| Low paternal education^¶^ | 28 (20) |
| ^*^Defined as presence or absence of siblings; ^$^defined as self-reported or doctor-diagnosed asthma; ^#^defined if one of the following was present: allergic asthma, allergic rhinitis, atopic dermatitis; ^+^positive in case of hives bigger than positive control histamine in any of the tested allergens; ^§^maternal active smoking during pregnancy; ^¶^parental education was categorised into low (<4 years of apprenticeship) and high (≥4 years of apprenticeship). Data are derived from n=142 subjects. | |

| **Table S2 Characteristics and prevalence of the SNPs employed in this study** | | | | | | |
| --- | --- | --- | --- | --- | --- | --- |
| **SNP** | **Location** | **Allele** | **MAF** | **HW *P*-value** | **Genotype distribution** | |
|  |  | (major/minor) | (%) |  | all | prevalence n (%) |
| rs880633 | missense | G/A | 46.2 | 0.564 | GG | 44 (30.9) |
|  |  |  |  |  | GA | 71 (50) |
|  |  |  |  |  | AA | 27 (19.1) |
| rs10399931 | upstream | G/A | 23.3 | 0.688 | GG | 85 (59.9) |
|  |  |  |  |  | GA | 52 (36.6) |
|  |  |  |  |  | AA | 5 (3.5) |
| rs10399805 | upstream | C/T | 10.5 | 1 | CC | 113 (79.6) |
|  |  |  |  |  | CT | 28 (19.7) |
|  |  |  |  |  | TT | 2 (1) |
| rs1538372 | intron | G/A | 33.8 | 1 | GG | 65 (45.8) |
|  |  |  |  |  | GA | 62 (43.7) |
|  |  |  |  |  | AA | 15 (10.6) |
| rs10920579^§^ | intergenic | C/T | 18.2 | 0.336 | CC | 92 (64.8) |
|  |  |  |  |  | CT | 49 (34.5) |
|  |  |  |  |  | TT | 1 (1) |
| rs7542294 | intron | C/T | 13.5 | 0.760 | CC | 108 (76.1) |
|  |  |  |  |  | CT | 31 (21.8) |
|  |  |  |  |  | TT | 3 (2.1) |
| rs2486064 | intergenic | C/T | 40.1 | 0.236 | CC | 50 (35.2) |
|  |  |  |  |  | CT | 64 (45.1) |
|  |  |  |  |  | TT | 28 (19.7) |
| HW: Hardy-Weinberg; MAF: minor allele frequency; SNP: single nucleotide polymorphism. ^§^Proxy for rs4950928: rs10920579 (r^2^=1, D’=1, calculated with HapMap). SNPs were genotyped with Illumina HumanOmniExpress Bead Chips. Data are derived from n=142 subjects. | | | | | | |

| **Table S3 Associations between SNPs and cord blood YKL-40 levels** | | | | | | | |
| --- | --- | --- | --- | --- | --- | --- | --- |
|  | Univariable model | | |  | Adjusted**^#^** model | | |
|  | Coef | 95% CI | *P*-value |  | Coef | 95% CI | *P*-value |
| ^$^SNP |  |  |  |  |  |  |  |
| rs10920579 | 5.40 | -5.07–15.87 | 0.976 |  | 5.91 | -4.75–16.57 | 0.936 |
| rs880633 | 3.57 | -4.13–11.27 | 0.976 |  | 3.64 | -4.11–11.38 | 0.936 |
| rs10399931 | 7.42 | -1.96–16.82 | 0.839 |  | 7.86 | -1.61–17.32 | 0.721 |
| rs10399805 | -0.18 | -13.05–15.5 | 0.976 |  | -0.53 | -13.56–12.51 | 0.936 |
| rs1538372 | 3.38 | -4.78–11.57 | 0.976 |  | 3.63 | -4.55–11.82 | 0.936 |
| rs7542294 | 3.26 | -8.01–14.55 | 0.976 |  | 3.02 | -8.37–14.21 | 0.936 |
| rs2486064 | 3.63 | -3.78–11.05 | 0.976 |  | 3.50 | -3.51–11.11 | 0.936 |
| Data is given as the probability of cord blood YKL-40 levels (ng/ml) being above detection per each SNP. Coef: Coefficient; CI: confidence interval; SNP: single nucleotide polymorphism. **^#^**This model was adjusted for sex, presence or absence of siblings, type of delivery, maternal atopy and parental education. ^$^*P-*values for SNPs are shown after correction for multiple testing according to Benjamini-Hochberg. Data are derived from n=142 subjects. | | | | | | | |

| **Table S4 Associations between SNPs and cord blood YKL-40 levels with weeks with any respiratory symptoms and weeks with severe respiratory symptoms during the first year of life** | | | | | | | | | | | |
| --- | --- | --- | --- | --- | --- | --- | --- | --- | --- | --- | --- |
|  | Univariable model | | |  | Multivariable^$^ model | | |  | Full Multivariable^*^ model | | |
|  | IRR^¶^ | 95% CI | *P*-value |  | IRR^¶^ | 95% CI | *P*-value |  | IRR^¶^ | 95% CI | *P*-value |
| Exposure | Outcome weeks with any respiratory symptoms**^#^** | | | | | | | | | | |
| SNP^#^ |  |  |  |  |  |  |  |  |  |  |  |
| rs10920579 | 0.97 | 0.73–1.23 | 0.731 |  | 0.96 | 0.76–1.19 | 0.963 |  | 0.98 | 0.79–1.19 | 0.994 |
| rs880633 | 1.12 | 0.92–1.35 | 0.731 |  | 1.09 | 0.91–1.29 | 0.963 |  | 1.09 | 0.92–1.29 | 0.994 |
| rs10399931 | 1.00 | 0.82–1.30 | 0.731 |  | 1.01 | 0.83–1.22 | 0.963 |  | 1.03 | 0.86–1.23 | 0.994 |
| rs10399805 | 1.34 | 0.97–1.75 | 0.512 |  | 1.25 | 0.96–1.62 | 0.592 |  | 1.25 | 0.99–1.58 | 0.364 |
| rs1538372 | 1.01 | 0.85–1.25 | 0.731 |  | 1.00 | 0.84–1.18 | 0.963 |  | 1.00 | 0.85–1.16 | 0.994 |
| rs7542294 | 1.21 | 0.92–1.53 | 0.731 |  | 1.17 | 0.92–1.47 | 0.963 |  | 1.21 | 0.97–1.49 | 0.428 |
| rs2486064 | 0.89 | 0.74–1.02 | 0.512 |  | 0.84 | 0.84–0.98 | 0.232 |  | 0.85 | 0.73–0.99 | 0.267 |
| YKL-40 (ng/ml) |  |  |  |  |  |  |  |  |  |  |  |
| YKL-40 non-detects | 1 | reference | *P*_trend_ |  | 1 | reference | *P*_trend_ |  | 1 | reference | *P*_trend_ |
| YKL-40 (7–37.9) | 1.05 | 0.5–2.1 |  |  | 1.22 | 0.6–2.4 |  |  | 1.16 | 0.5–2.3 |  |
| YKL-40 (38–49.9) | 1.25 | 0.5–2.8 |  |  | 1.43 | 0.6–3.2 |  |  | 1.39 | 0.6–3.1 |  |
| YKL-40 (50–65.9) | 1.13 | 0.6–2.3 |  |  | 1.31 | 0.6–2.6 |  |  | 1.48 | 0.7–2.9 |  |
| YKL-40 (66–98) | 1.42 | 0.7–2.7 | 0.131^£^ |  | 1.38 | 0.7–2.5 | 0.127^£^ |  | 1.40 | 0.7–2.6 | 0.237^£^ |
|  |  |  |  |  |  |  |  |  |  |  |  |
| Exposure | Outcome weeks with severe respiratory symptoms**^§^** | | | | | | | | | | |
| SNP^#^ |  |  |  |  |  |  |  |  |  |  |  |
| rs10920579 | 1.23 | 0.79–1.91 | 0.571 |  | 1.20 | 0.81–1.77 | 0.969 |  | 1.20 | 0.82–1.75 | 0.820 |
| rs880633 | 1.16 | 0.76–1.74 | 0.571 |  | 1.07 | 0.72–1.57 | 0.969 |  | 1.09 | 0.74–1.59 | 0.820 |
| rs10399931 | 1.20 | 0.81–1.74 | 0.571 |  | 1.16 | 0.82–1.63 | 0.969 |  | 1.22 | 0.86–1.71 | 0.820 |
| rs10399805 | 1.44 | 0.89–2.29 | 0.571 |  | 1.31 | 0.73–2.03 | 0.969 |  | 1.27 | 0.82–1.93 | 0.820 |
| rs1538372 | 1.06 | 0.74–1.51 | 0.571 |  | 1.01 | 0.84–1.38 | 0.969 |  | 1.04 | 0.77–1.38 | 0.820 |
| rs7542294 | 1.23 | 0.76–1.98 | 0.571 |  | 1.19 | 0.73–1.94 | 0.969 |  | 1.21 | 0.73–1.96 | 0.820 |
| rs2486064 | 0.93 | 0.65–1.30 | 0.571 |  | 0.88 | 0.63–1.21 | 0.969 |  | 0.90 | 0.67–1.21 | 0.820 |
| YKL-40 (ng/ml) |  |  |  |  |  |  |  |  |  |  |  |
| YKL-40 non-detects | 1 | reference | *P*_trend_ |  | 1 | reference | *P*_trend_ |  | 1 | reference | *P*_trend_ |
| YKL-40 (7–37.9) | 0.93 | 0.47–1.83 |  |  | 1.13 | 0.59–2.18 |  |  | 1.06 | 0.54–2.06 |  |
| YKL-40 (38–49.9) | 1.13 | 0.49–2.56 |  |  | 1.36 | 0.63–2.95 |  |  | 1.28 | 0.61–2.74 |  |
| YKL-40 (50–65.9) | 1.08 | 0.55–2.09 |  |  | 1.25 | 0.66–2.39 |  |  | 1.41 | 0.73–2.71 |  |
| YKL-40 (66–98) | 1.27 | 0.67–2.41 | 0.372^£^ |  | 1.29 | 0.67–2.46 | 0.234^£^ |  | 1.32 | 0.71–2.51 | 0.190^£^ |
| CI: confidence interval; IRR: incidence risk ratio; SNP: single nucleotide polymorphism. ^$^Adjusted for sex, gestational weight, maternal smoking during pregnancy and maternal atopic disease. ^*^Adjusted for sex, gestational weight, maternal smoking during pregnancy, maternal atopic disease, presence or absence of siblings, nursery, parental smoking during the first year, parental education. **^#^**Defined as any (day or night) respiratory symptoms independent of severity. **^§^**Defined as any (day or night) respiratory symptoms with a symptom score $\geq$3 [[3](#_ENREF_3)]. ^#^*P-*values for SNPs are shown after correction for multiple testing according to Benjamini-Hochberg. ^£^*P*_trend_ -values were calculated with the Cochran-Armitage trend test. Data are derived from n=142 subjects. | | | | | | | | | | | |

| **Table S5 Associations between SNPs and cord blood YKL-40 levels with lung function at 5 weeks** | | | | | | | |
| --- | --- | --- | --- | --- | --- | --- | --- |
|  | Univariable association | | |  | Adjusted^*^ association | | |
|  | Coef | 95% CI | *P*-value |  | Coef | 95% CI | *P*-value |
| Exposure |  | Outcome tidal volume (ml) ^$^ | | | | | |
| SNP^#^ |  |  | | | | | |
| rs10920579 | 0.96 | 0.91–1.01 | 0.793 |  | 0.96 | 0.91–1.01 | 0.541 |
| rs880633 | 0.99 | 0.94–1.03 | 0.793 |  | 0.98 | 0.94–1.03 | 0.920 |
| rs10399931 | 0.96 | 0.91–1.01 | 0.793 |  | 0.96 | 0.91–1.01 | 0.449 |
| rs10399805 | 1.01 | 0.94–1.08 | 0.793 |  | 1.00 | 0.94–1.08 | 0.920 |
| rs1538372 | 0.97 | 0.93–1.01 | 0.793 |  | 0.98 | 0.93–1.01 | 0.920 |
| rs7542294 | 1.01 | 0.95–1.07 | 0.793 |  | 1.01 | 0.95–1.07 | 0.920 |
| rs2486064 | 0.99 | 0.95–1.03 | 0.793 |  | 0.98 | 0.95–1.03 | 0.920 |
| YKL-40 (ng/ml) |  |  |  |  |  |  |  |
| YKL-40 non-detects | 1 | reference | *P*_trend_ |  | 1 | reference | *P*_trend_ |
| YKL-40 (7–37.9) | 1.05 | 0.97–1.14 |  |  | 1.02 | 0.87–1.18 |  |
| YKL-40 (38–49.9) | 0.95 | 0.89–1.03 |  |  | 0.98 | 0.85–-1.13 |  |
| YKL-40 (50–65.9) | 1.00 | 0.99–1.08 |  |  | 1.05 | 0.91–1.21 |  |
| YKL-40 (66–98) | 1.00 | 0.92–1.08 | 0.930^£^ |  | 0.98 | 0.85–1.13 | 0.841^£^ |
|  |  |  |  |  |  |  |  |
| Exposure |  | Outcome mean tidal expiratory flow^$^ | | | | | |
| SNP^#^ |  |  | | | | | |
| rs10920579 | 1.05 | 0.97–1.13 | 0.41 |  | 1.05 | 0.98–1.13 | 0.478 |
| rs880633 | 1.03 | 0.98–1.08 | 0.41 |  | 1.03 | 0.98–1.08 | 0.478 |
| rs10399931 | 0.94 | 1.01–1.14 | 0.178 |  | 1.07 | 1.07–1.14 | 0.233 |
| rs10399805 | 1.06 | 0.86–1.03 | 0.41 |  | 0.94 | 0.86–1.03 | 0.478 |
| rs1538372 | 1.07 | 1.01–1.12 | 0.112 |  | 1.06 | 1.01–1.12 | 0.227 |
| rs7542294 | 0.93 | 0.86–1.01 | 0.41 |  | 0.93 | 0.86–1.01 | 0.431 |
| rs2486064 | 1.02 | 0.97–1.07 | 0.41 |  | 1.02 | 0.97–1.07 | 0.478 |
| YKL-40 (ng/ml |  |  |  |  |  |  |  |
| YKL-40 non-detects | 1 | reference | *P*_trend_ |  | 1 | reference | *P*_trend_ |
| YKL-40 (7–37.9) | 0.99 | 0.88–1.11 |  |  | 0.983 | 0.88–1.09 |  |
| YKL-40 (38–49.9) | 1.05 | 0.94–1.17 |  |  | 1.06 | 0.95–1.17 |  |
| YKL-40 (50–65.9) | 1.05 | 0.94–1.17 |  |  | 1.06 | 0.95–1.17 |  |
| YKL-40 (66–98) | 1.03 | 0.93–1.15 | 0.152^£^ |  | 1.03 | 0.93–1.14 | 0.194^£^ |
|  |  |  |  |  |  |  |  |
| Exposure |  | Outcome ratio T_PTEF_/T_E_%^$^ | | | | | |
| SNP^#^ |  |  | | | | | |
| rs10920579 | 1.10 | 0.98–1.21 | 0.549 |  | 1.08 | 0.97–1.19 | 0.959 |
| rs880633 | 0.96 | 0.89–1.03 | 0.999 |  | 0.96 | 0.89–1.04 | 0.971 |
| rs10399931 | 1.04 | 0.94–1.14 | 0.999 |  | 1.03 | 0.93–1.13 | 0.971 |
| rs10399805 | 0.98 | 0.86–1.11 | 0.999 |  | 0.99 | 0.86–1.12 | 0.971 |
| rs1538372 | 1.00 | 0.91–1.07 | 0.999 |  | 0.99 | 0.91–1.06 | 0.971 |
| rs7542294 | 0.93 | 0.83–1.04 | 0.999 |  | 0.94 | 0.94–1.05 | 0.971 |
| rs2486064 | 1.00 | 0.93–1.07 | 0.999 |  | 1.00 | 0.99–1.07 | 0.971 |
| YKL-40 (ng/ml) |  |  |  |  |  |  |  |
| YKL-40 non-detects | 1 | reference | *P*_trend_ |  | 1 | reference | *P*_trend_ |
| YKL-40 (7–37.9) | 1.04 | 0.89–1.21 |  |  | 1.02 | 0.89–1.21 |  |
| YKL-40 (38–49.9) | 1.00 | 0.86–1.15 |  |  | 0.98 | 0.86–1.15 |  |
| YKL-40 (50–65.9) | 1.06 | 0.91–1.12 |  |  | 1.05 | 0.91–1.12 |  |
| YKL-40 (66–98) | 0.99 | 0.85–1.14 | 0.866^£^ |  | 0.98 | 0.85–1.14 | 0.818^£^ |
|  |  |  |  |  |  |  |  |
| Exposure |  | Outcome minute ventilation (ml · min^-1^) ^$^ | | | | | |
| SNP^#^ |  |  | | | | | |
| rs10920579 | 1.03 | 0.97–1.09 | 0.409 |  | 1.02 | 0.96–1.08 | 0.507 |
| rs880633 | 1.04 | 0.99–1.08 | 0.409 |  | 1.03 | 0.98–1.07 | 0.507 |
| rs10399931 | 1.05 | 0.99–1.11 | 0.395 |  | 1.04 | 0.99–1.09 | 0.507 |
| rs10399805 | 0.96 | 0.89–1.03 | 0.409 |  | 0.96 | 0.90–1.02 | 0.507 |
| rs1538372 | 1.06 | 1.01–1.11 | 0.053 |  | 1.05 | 1.01–1.09 | 0.114 |
| rs7542294 | 0.95 | 0.89–1.01 | 0.409 |  | 0.96 | 0.91–1.01 | 0.507 |
| rs2486064 | 1.03 | 0.96–1.05 | 0.409 |  | 1.01 | 0.97–1.05 | 0.507 |
| YKL-40 (ng/ml) |  |  |  |  |  |  |  |
| YKL-40 non-detects | 1 | reference | *P*_trend_ |  | 1 | reference | *P*_trend_ |
| YKL-40 (7–37.9) | 1.01 | 0.93–1.11 |  |  | 1.00 | 0.92–1.09 |  |
| YKL-40 (38–49.9) | 1.03 | 0.95–1.12 |  |  | 1.03 | 0.94–1.11 |  |
| YKL-40 (50–65.9) | 1.04 | 0.96–1.14 |  |  | 1.05 | 0.96–1.13 |  |
| YKL-40 (66–98) | 1.05 | 0.96–1.14 | 0.174^£^ |  | 1.04 | 0.95–1.12 | 0.230^£^ |
| Coef: coefficient; CI: confidence interval; T_PTEF_/T_E_ ratio of time to reach peak tidal expiratory flow to total expiratory time. All lung function parameters were log converted for analysis and results shown as exponentiated coefficients. ^*^Adjusted for the following additional risk factors: sex, gestational length, postnatal age at lung function, weight at lung function. ^$^Missing data on n=20; data available for n=122.  ^#^*P-*values for SNPs are shown after correction for multiple testing according to Benjamini-Hochberg. ^£^*P*_trend_ -values were calculated with the Cochran-Armitage trend test. | | | | | | | |

| **Table S6 Associations between SNPs and cord blood YKL-40 levels with positive prick test at school age** | | | | | | | |
| --- | --- | --- | --- | --- | --- | --- | --- |
|  | Univariable association | | |  | Multivariable^*^ association | | |
|  | OR | 95% CI | *P*-value |  | OR | 95% CI | *P*-value |
| Exposure | Outcome positive prick test^+^ | | | | | | |
| SNP^#^ |  | | | | | | |
| rs10920579 | 0.78 | 0.27–2.19 | 0.921 |  | 0.65 | 0.22–1.90 | 0.983 |
| rs880633 | 0.96 | 0.46–1.99 | 0.921 |  | 0.87 | 0.39–1.88 | 0.983 |
| rs10399931 | 0.56 | 0.21–1.14 | 0.921 |  | 0.46 | 0.17–1.12 | 0.945 |
| rs10399805 | 0.89 | 0.24–3.19 | 0.921 |  | 0.99 | 0.26–3.36 | 0.983 |
| rs1538372 | 1.06 | 0.49–2.23 | 0.921 |  | 0.93 | 0.42–2.01 | 0.983 |
| rs7542294 | 0.91 | 0.31–2.64 | 0.921 |  | 0.92 | 0.32–2.69 | 0.983 |
| rs2486064 | 0.93 | 0.45–1.91 | 0.921 |  | 0.78 | 0.36–1.63 | 0.983 |
|  |  |  |  |  |  |  |  |
| YKL-40 (ng/ml) |  |  |  |  |  |  |  |
| YKL-40 non-detects | 1 | reference | *P*_trend_ |  | 1 | reference | *P*_trend_ |
| YKL-40 (7–37.9) | 1.01 | 0.24–4.24 |  |  | 0.95 | 0.22–4.14 |  |
| YKL-40 (38–49.9) | 1.24 | 0.29–5.26 |  |  | 1.05 | 0.23–4.73 |  |
| YKL-40 (50–65.9) | 1.17 | 0.27–4.96 |  |  | 1.07 | 0.25–4.64 |  |
| YKL-40 (66–98) | 2.8 | 0.75–10.42 | 0.128^£^ |  | 2.56 | 0.66–9.88 | 0.169^£^ |
| CI: confidence interval; OR: odds ratio; SNP: single nucleotide polymorphism. ^*^Adjusted for the following additional risk factors: sex, parental smoking during childhood, maternal atopy, parental education. ^+^Positive in case of hives bigger than positive control histamine in any of the tested allergens. Missing data on n=38; data available for n=104. ^#^*P-*values for SNPs are shown after correction for multiple testing according to Benjamini-Hochberg. ^£^*P*_trend_-values were calculated with the Cochran-Armitage trend test. | | | | | | | |

| **Table S7 Associations between SNPs and cord blood YKL-40 levels with lung function at 6 years** | | | | | | | |
| --- | --- | --- | --- | --- | --- | --- | --- |
|  | Univariable association | | |  | ^§^Adjusted association | | |
|  | Coef | 95% CI | p-value |  | Coef | 95% CI | p-value |
| Exposure | Outcome FVC (z-score)^$^ | | | | | | |
| SNP^#^ |  |  |  |  |  |  |  |
| rs10920579 | 0.27 | -0.29–0.47 | 0.803 |  | 0.10 | -0.31–0.51 | 0.968 |
| rs880633 | 0.18 | -0.07–0.44 | 0.609 |  | 0.17 | -0.10–0.44 | 0.968 |
| rs10399931 | 0.05 | -0.29–0.38 | 0.803 |  | 0.05 | -0.29–0.49 | 0.968 |
| rs10399805 | 0.44 | -0.06–0.95 | 0.526 |  | 0.44 | -0.11–1.02 | 0.714 |
| rs1538372 | 0.01 | -0.26 –0.29 | 0.803 |  | -0.01 | -0.30–0.29 | 0.968 |
| rs7542294 | 0.43 | 0.01–0.85 | 0.332 |  | 0.43 | -0.01–0.84 | 0.336 |
| rs2486064 | -0.14 | -0.44–0.15 | 0.803 |  | -0.15 | -0.44–0.13 | 0.968 |
| YKL-40 (ng/ml) |  |  |  |  |  |  |  |
| YKL-40 non-detects | 1 | reference | *P*_trend_ |  | 1 | reference | *P*_trend_ |
| YKL-40 (7–37.9) | 0.14 | -0.44–0.72 |  |  | 0.20 | -0.40–0.81 |  |
| YKL-40 (38–49.9) | 0.16 | -0.43–0.74 |  |  | 0.21 | -0.41–0.82 |  |
| YKL-40 (50–65.9) | 0.10 | -0.45–0.65 |  |  | 0.16 | -0.43–0.75 |  |
| YKL-40 (66–98) | 0.34 | -0.22–0.91 | 0.280^£^ |  | 0.39 | -0.21–0.99 | 0.326^£^ |
|  |  |  |  |  |  |  |  |
| Exposure | Outcome FEV_1_  (z-score) ^#^ | | | | | | |
| SNP^#^ |  |  | | | | | |
| rs10920579 | -0.07 | -0.47–0.33 | 0.743 |  | -0.09 | -0.52–0.33 | 0.863 |
| rs880633 | 0.05 | -0.24–0.33 | 0.743 |  | 0.03 | -0.22–0.25 | 0.863 |
| rs10399931 | -0.08 | -0.44–0.28 | 0.743 |  | -0.09 | -0.61–0.25 | 0.863 |
| rs10399805 | 0.36 | -0.15–0.29 | 0.743 |  | 0.36 | -0.14–0.22 | 0.863 |
| rs1538372 | -0.14 | 0.45–0.86 | 0.743 |  | -0.17 | 0.44–0.19 | 0.863 |
| rs7542294 | 0.26 | -0.41–0.17 | 0.743 |  | 0.27 | -0.15–0.67 | 0.863 |
| rs2486064 | -0.23 | -0.52–0.67 | 0.743 |  | -0.26 | -0.53–0.07 | 0.721 |
| YKL-40 (ng/ml) |  |  |  |  |  |  |  |
| YKL-40 non-detects | 1 | reference | *P*_trend_ |  | 1 | reference | *P*_trend_ |
| YKL-40 (7–37.9) | 0.01 | -0.59–0.61 |  |  | 0.09 | -0.53–0.71 |  |
| YKL-40 (38–49.9) | -0.14 | -0.73–0.45 |  |  | -0.04 | -0.67–0.61 |  |
| YKL-40 (50–65.9) | 0.03 | -0.54–0.61 |  |  | 0.11 | -0.51–0.73 |  |
| YKL-40 (66–98) | 0.28 | -0.31–0.88 | 0.428^£^ |  | 0.43 | -0.21–1.11 | 0.239^£^ |
|  |  |  |  |  |  |  |  |
| Exposure | Outcome FEF_25-75%_  (z-score)^&^ | | | | | | |
| SNP^#^ |  |  | | | | | |
| rs10920579 | -0.10 | -0.51–0.31 | 0.963 |  | -0.18 | -0.61–0.23 | 0.709 |
| rs 880633 | -0.30 | -0.58–0.27 | 0.834 |  | -0.30 | -0.57–0.01 | 0.276 |
| rs10399931 | -0.17 | -0.52–0.19 | 0.963 |  | -0.22 | -0.59–0.13 | 0.709 |
| rs10399805 | -0.18 | -0.72–0.36 | 0.963 |  | -0.11 | -0.66–0.43 | 0.709 |
| rs1538372 | -0.29 | -0.58–0.01 | 0.963 |  | -0.31 | -0.58–0.01 | 0.332 |
| rs7542294 | -0.16 | -0.61–0.29 | 0.963 |  | -0.15 | -0.62–0.28 | 0.709 |
| rs2486064 | -0.13 | -0.44–0.18 | 0.963 |  | -0.14 | -0.46–0.17 | 0.709 |
| YKL-40 (ng/ml) |  |  |  |  |  |  |  |
| YKL-40 non-detects | 1 | reference | *P*_trend_ |  | 1 | reference | *P*_trend_ |
| YKL-40 (7–37.9) | 0.25 | -0.31–0.81 |  |  | 0.27 | -0.31–0.84 |  |
| YKL-40 (38–49.9) | -0.26 | -0.83–0.31 |  |  | -0.17 | -0.77–0.43 |  |
| YKL-40 (50–65.9) | -0.08 | -0.61–0.45 |  |  | -0.05 | -0.61–0.52 |  |
| YKL-40 (66–98) | -0.07 | -0.61–0.47 | 0.610^£^ |  | 0.01 | -0.56–0.59 | 0.836^£^ |
| Coef: Coefficient; CI: confidence interval; SNP: single nucleotide polymorphism; FVC: forced vital capacity; FEV_1_: forced expiratory volume in one second; FEF: forced expiratory flow from 25-75% of exhalation. ^$^Missing data on n=51; data available for n=91. ^#^Missing data on n=66; data available for n=76. ^&^Missing data on n=62; data available for n=80. ^§^Adjusted for presence or absence of siblings, daycare attendance and asthma of the child. ^#^*P-*values for SNPs are shown after correction for multiple testing according to Benjamini-Hochberg.  ^£^ *P*_trend_ -values were calculated with the Cochran-Armitage trend test. | | | | | | | |
